# Aversive view memories and risk perception in navigating ants

**DOI:** 10.1038/s41598-022-06859-4

**Published:** 2022-02-21

**Authors:** Cody A. Freas, Antoine Wystrach, Sebastian Schwarz, Marcia L. Spetch

**Affiliations:** 1 Department of Psychology, University of Alberta, Edmonton, AB T6G 2R3 Canada; 2 Research Center On Animal Cognition (CRCA), Center for Integrative Biology (CBI), CNRS, University Toulouse III-Paul Sabatier, Toulouse, France

**Keywords:** Animal behaviour, Entomology, Behavioural ecology, Cognitive neuroscience, Learning and memory, Sensory processing, Visual system, Psychology

## Abstract

Many ants establish foraging routes through learning views of the visual panorama. Route models have focused primarily on attractive view use, which experienced foragers orient towards to return to known sites. However, aversive views have recently been uncovered as a key component of route learning. Here, *Cataglyphis velox* rapidly learned aversive views, when associated with a negative outcome, a period of captivity in vegetation, triggering increases in hesitation behavior. These memories were based on the accumulation of experiences over multiple trips with each new experience regulating forager hesitancy. Foragers were also sensitive to captivity time differences, suggesting they possess some mechanism to quantify duration. Finally, we analyzed foragers' perception of risky (i.e. variable) versus stable aversive outcomes by associating two sites along the route with distinct captivity schedules, a fixed or variable duration, with the same mean across training. Foragers exhibited fewer hesitations in response to risky outcomes compared to fixed ones, indicating they perceived risky outcomes as less severe. Results align with a logarithmic relationship between captivity duration and hesitations, suggesting that aversive stimulus perception is a logarithm of its actual value. We discuss how aversive view learning could be executed within the mushroom bodies circuitry following a prediction error rule.

## Introduction

The navigational abilities of solitarily foraging ants can be attributed to a toolkit comprised of multiple strategies^1–4^. The most well studied components of this toolkit are the path integration (PI) system^5–7^, which is useful when visual terrestrial cues are not available as well as during route formation^8–11^, and the learned visual cues of the panorama^12–17^. Many ant species rapidly learn visual landmark information to navigate while foraging and rather than learn individual landmarks, foragers acquire panoramic views around goal locations and along their foraging routes^12,13^. View learning first occurs around the nest during learning walks prior to the onset of foraging^14,15^. Foragers also acquire views while en-route as they move away from known locations^12,13,16,17^, and can rapidly learn the panorama at a new site, often after only one previous experience^15,18,19^. Foragers retain long term-memories of these panoramas^20^ and, while navigating, compare these memories to their current view to recover their goal direction^21^.

### Aversive views

View memories and their importance in route following have been well modeled^21–26^, yet these models rely principally on the forager orienting towards attractive views via view comparison. Recent work has expanded this modelling to include the learning of views that are repellent or aversive and cause foragers to turn away from views not associated with the current goal, resulting in orientation away from incorrect directions^27,28^. Interactions between these learned attractive and aversive views permit navigators to compare a single current view to their view memories to quickly decide whether to move toward or turn away from a given direction^27,28^. It has been suggested that learned views can become aversive depending on their orientation relative to the nest^28,29^, on the foraging motivational context^30^ or when these views are associated with aversive outcomes^31^. Additionally, a view memory’s valence can be altered, with previously attractive views becoming aversive when they are associated with negative outcomes. In both *Cataglyphis* and *Melophorus* desert ants, when a pit trap was added along a forager’s homeward route, resulting in foragers falling into dry vegetation, ants quickly memorized the views experienced just before this negative experience as aversive or repellent. Eventually, after a few experiences falling into this pit, foragers formed new routes detouring around it. The interplay of aversive and attractive views appears to facilitate the formation of these detours. As foragers attempt to avoid aversive views, novel views that pilot around these areas become positively reinforced, leading to the development of new routes detouring around obstacles and areas with difficult terrain^31^. Dense vegetation can often be hard for desert ants to move through effectively, especially when carrying food, resulting in increases to both expenditure of effort and the delay to return to the nest. After only a few trips experiencing the vegetation-filled pit, foragers began to hesitate near the pit’s edge, increasing their hesitancy to pass through the area, evidenced through increases in both scanning behavior and path meander^31^. Scanning behavior consists of a forager stopping forward movement and rotating their body on the spot. This behavior is associated with instances of increased navigational uncertainty: when the familiarity of the panorama decreases, the PI and panorama enter into conflict, or when the current route’s panorama is associated with failure^32,33^. Thus, the incidence of scanning is a good behavioral proxy to assess the ant’s uncertainty and here we used it, along with stopping behaviors, to quantify the strength of the aversion associated with a given location^30^.

### Risk perception

While navigating, foraging animals must make decisions assessing risky or safe options both in the resources they collect and in their foraging routes. Across a number of animal models, individuals will sometimes behave in seemingly non-optimal or irrational ways with regards to their perception and preference for risk^34–36^. For example, when risk is generated by variability in amount, animals are often risk-averse or risk neutral, whereas when risk is generated by variability in delay, animals are typically risk-prone^36,37^. These preferences are believed to flow from animals’ perception of the world, where true stimulus strength has a logarithmic relationship with the animal’s perception^37–40^. Based on this principle, animals’ choices between risky (variable) and fixed outcomes should be predicted not by the arithmetic mean of these options but instead by their geometric means.

Non-optimal responses to risk have been shown to occur across a range of animals, even in species which typically forage by collecting food to provide energy for a group or colony such as ants and bees^36,41^. Much of the risk preference research in Hymenoptera has focused on foragers’ preference for risk solely in regards to the amount or quality of a given reward^36,42,43^. In honeybees and bumble bees, a variety of outcomes have been reported with foragers showing evidence of no preference, risk avoidance and risk seeking foraging choices based on factors such as colony resource levels^44–47^. In ants, risk perception and sensitivity have been explored on the colony level, focusing on how collective decision-making influences choice in the assessment of potential nesting site quality and in food reward quality. Rock ants (*Temnothorax albipennis*) were shown to exhibit risk seeking behavioral choices when making collective choices between nests^48^. The collective decision-making of the colony has also been shown to result in the avoidance of certain irrational choice behaviors observed in individual ants, including reducing the time to choose between potential nest sites^49,50^. Recently, De Agrò and colleagues^43^ have shown that non-optimal risk preferences in foraging ants may stem from how they perceive stimulus strength, with perception of a given stimulus having a logarithmic relationship with the stimulus’ strength. The researchers showed that ant foragers’ perceptions of food reward quality were on a logarithmic scale. Individuals were risk averse and preferred the fixed option when choosing between food rewards with the same mean values, however this preference disappeared when they chose between two logarithmically balanced alternatives^43^.

In the current study, we characterized foragers’ learning and memories of aversive views when these views are associated with aversive, high effort outcomes, i.e. being kept within a vegetation-filled vial for set time periods. Forcing foragers into vegetation simulates areas along the homeward route that contain dense clutter, compelling the forager to struggle through in order to reach the nest with its food piece, increasing both their time and energy expenditure. Here, we used a similar negative outcome to the previously described pit trap experiment^31^ with individual foragers struggling through vegetation before returning to the homebound route. Holding foragers within a vial instead of using the pit trap allowed us to more easily control hold time durations and prevented foragers from forming routes that avoid the negative outcome. Foragers’ behaviors were recorded using a trial-by-trial approach to describe navigational learning during natural tasks^51^. We first studied the dynamics of view learning, as well as retention across non-reinforced trials. Second, we explored foragers’ perception of captivity duration by training foragers to associate sites along the route with two distinct fixed durations of captivity (15 s vs. 300 s). Finally, we characterized foragers’ perception of risk when sites were associated with ‘Fixed’ or ‘Risky’ outcomes with the same mean duration across training (~ 150 s). Here, the ‘Fixed’ outcome was associated with a constant period held in vegetation (150 s) while the ‘Risky’ outcome was associated with a variable time period where foragers had a 50/50 chance on each trip of being held within vegetation for either 1 s or 300 s. We found that *C. velox* foragers rapidly learned to associate the (previously positive) homeward route views with aversive outcomes, with as few as two prior experiences. These aversive view memories persisted over multiple trips after the outcome was removed. Foragers were able to perceive differences in outcome severity, learning more rapidly and exhibiting more overall hesitations to views associated with more severe outcomes (300 s) compared to less severe outcomes (15 s). Finally, foragers showed significantly less apprehension to travel through sites associated with risky aversive outcomes compared to a fixed outcome with the same mean, suggesting non-linear scaling of captivity duration. The observed forager hesitations at the risky site were in line with the geometric average of the captivity durations, suggesting that the perception of aversive stimulus strength is logarithmic.

## Methods

### Study site and species

Testing was conducted in June and July 2019 on a single *C. velox* nest located at an established field site ~ 6 km south of Seville, Spain (37°19′51″N, 5°59′23″W). *C. velox* inhabit visually cluttered semi-arid environments, densely covered in grass tussocks, scattered bushes and with distant stands of trees and man-made structures. Foraging *C. velox* travel between sites alone and do not rely on pheromone trails while foraging. While navigating, these foragers rely heavily on these visual cues to return to the nest and known food sites, creating stable routes between locations^10,11^. There are no institutional or governmental regulations (in Canada or Spain) for research in invertebrates, manipulations were non-invasive and all individuals were returned to the nest after testing.

### Testing arena

**Figure 1:**
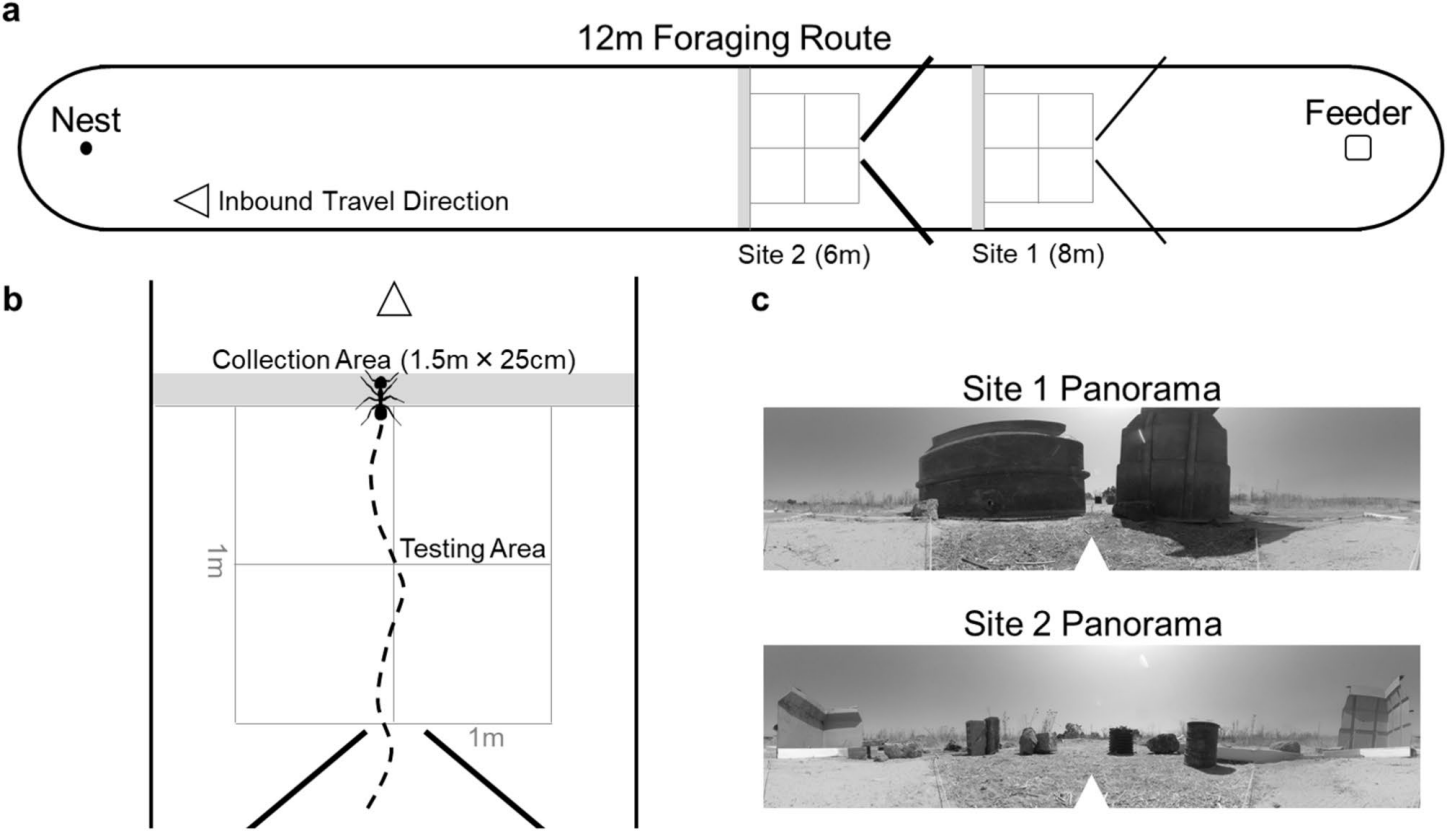
Diagram of the experimental set-up in all conditions. (**a**) Foragers were allowed to travel freely on the outbound trip to the feeder and collect food. After their release from the feeder, foragers travelled back to the nest through the testing areas at Site 1 and Site 2 and were collected based on condition. (**b**) Foragers were collected within a 25 cm (grey) area after passing 8 m (Site 1) or 6 m (Site 2) from the nest to allow the researcher to remain as far back as possible prior to collection. After the allotted hold period, the vial was placed at the centre of the collection area and foragers were allowed to climb out and resume their homeward trip. The testing areas were arranged with blocking walls to both funnel foragers to the centre of the arena as they reached each testing area as well as to create distinct visual panoramas at each site. Nest and inbound travel direction is denoted by the arrow. (**c**) Panoramic 360° photos of the surrounding visual cues at each collection site. In each photo, the white arrow denotes the nest direction.

A plastic square-shaped feeder (15 cm × 15 cm × 8 cm) was sunk into the ground 12 m from the nest entrance and was continuously stocked with crushed cookie pieces (Royal Dansk™). The smooth walls of the feeder prevented foragers that dropped in from exiting without being lifted out by the researcher. All vegetation in a 200 cm wide band from the nest to the feeder and in a 100 cm radius around both sites was removed using an edge trimmer. To entice foragers to collect food only from the feeder, an arena was erected using a 10 cm high smooth plastic barrier, enclosing the nest and feeder site and restricting the nest to forage only within the arena. This arena was 150 cm in width and extended in a 75 cm radius semi-circle around both feeder and nest (Fig. 1a). Two collection sites along the feeder-nest route were designated at 8 m and 6 m (Site 1 and Site 2 respectively) from the nest. To record inbound forager behaviour leading up to each site, two grids consisting of 2 × 2 of 50 cm squares were erected using string and metal pegs extending from each collection site 100 cm towards the feeder (ending at 9 m for Site 1 and 7 m for Site 2; Fig. 1a,b). Two sets of barriers were erected at 45º angles creating a ~ 20 cm gap at the edge of the grid to funnel foragers toward the centre of the arena’s width (Fig. 1a,b). To create two distinct panoramic scenes, at Site 1 the first set of erected barriers were 10 cm high plastic walls identical to the walls of the arena, while the second set of barriers leading to Site 2 were 120 cm high (Fig. 1c). Additionally, to increase the panorama differences between sites, we placed a number of shorter 15–25 cm visual landmarks consisting of stones and bricks around Site 2 (See Fig. 1c). This arena set-up was used for all three experiments.

Upon the completion of the foraging arena construction, we allowed the nest two days to discover the feeder and begin consistently foraging. For these 2 days, any foragers reaching the feeder were allowed to enter and exit via a wooden ramp. At the onset of training this ramp was removed. As foraging began on the third day, it was expected foragers used in the experiments had some level of knowledge of the route before the onset of training and had learned the positive association between the views of the route and successful foraging trips. While the exact level of experience with the route prior to training may have individually varied, forager experience of the route during training was strictly controlled. When a researcher was not present to conduct training/testing, all foragers were restricted to a 20 cm area around the nest using a plastic cylinder (~ 20 cm in height).

### Procedure

#### Aversive learning tests

**Figure 2:**
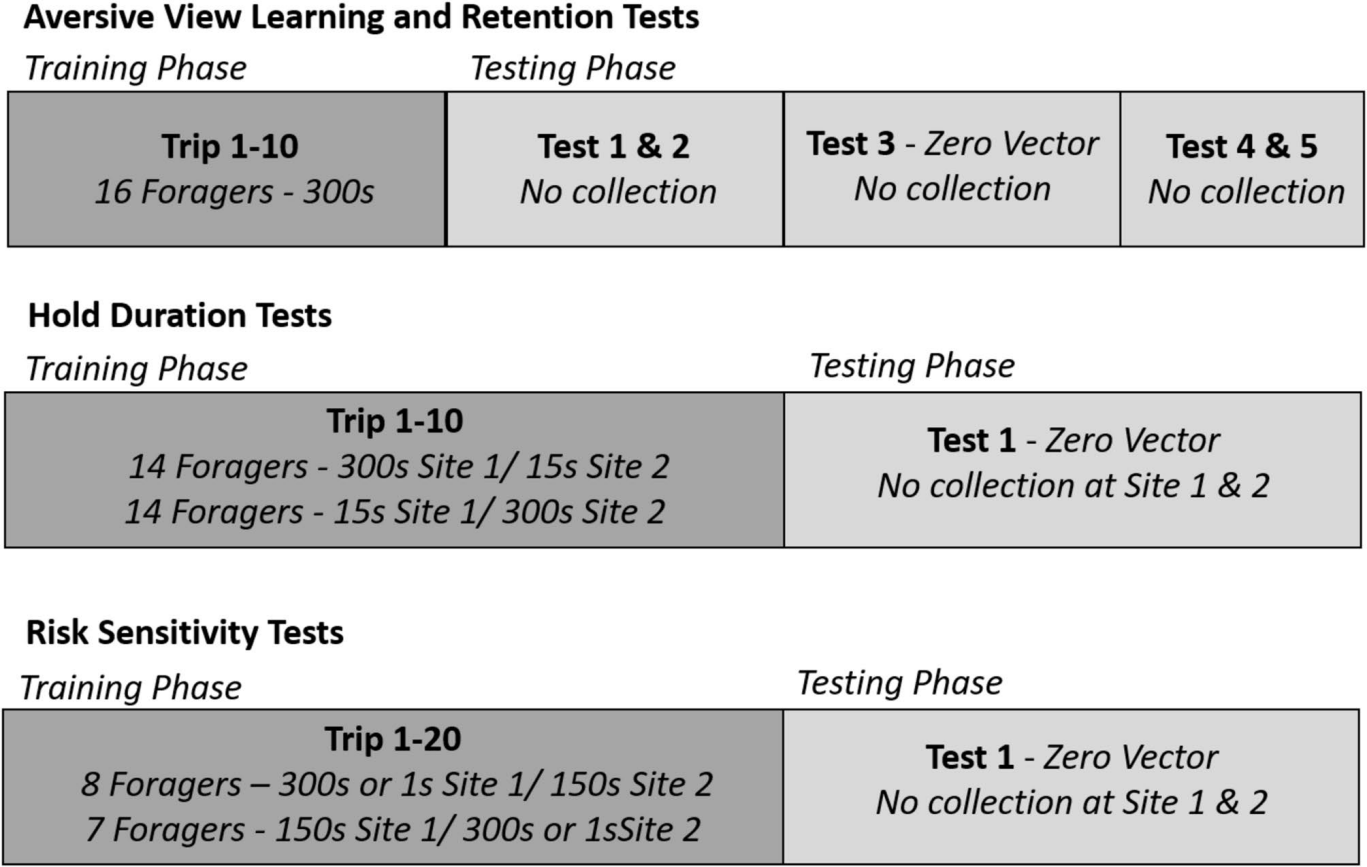
Timeline of training and testing procedures in all conditions.

We initially tested foragers’ learning of aversive view memories by collecting inbound foragers as they reached Site 2 (Figs. 1a, 2). When approaching a view that has been associated with a negative outcome, foragers have been shown to hesitate leading up to the site, exhibiting bouts of scanning behaviour as well as attempting to avoid these sites via detours^31^. In the current study, we collected two types of hesitation behaviour, scans and stops. Scans were defined as the ceasing of forager movement that was accompanied by the forager clearly turning on the spot, rotating in place. In contrast, stops were cataloged as the ceasing of forward movement with no accompanying rotation.

During training, each forager (n = 16) was exposed to ten consecutive training trips, followed by five tests (Fig. 2). At the onset of training, foragers were allowed to travel freely from the nest to the feeder. Once in the feeder, foragers were individually marked using enamel paint (Tamiya), returned to the feeder and allowed to collect a cookie piece. After collecting food, foragers were lifted out of the feeder by hand and allowed to travel through the arena to the nest. As foragers neared the grid at Site 2, their paths were recorded using an HD camera at 30 fps with a 3840 × 2160 pixels image size (GoPro) positioned 120 cm above the grid facing down. Recording started just before the forager entered the grid area and ceased at collection. As the forager exited the grid (6 m from the nest entrance), they were exposed to an aversive outcome in which they were held captive in a vegetation-filled vial. Specifically, foragers were collected within a 25 cm area (grey area, Fig. 1a,b) using an opaque plastic 5 cm diameter vial, which was filled with ~ 10 cm of loosely packed grass from the surrounding vegetation, and held within this vegetation for a period of 300 s. During this holding period, the vial was covered and placed in a semi-shaded area to prevent overheating. Additionally, the lid of this vial rested lightly upon the top of the vegetation, preventing foragers from standing on top of the vegetation during their hold period. After 300 s, the vial was placed at the center of Site 2’s collection area (grey area, Fig. 1b) and tilted ~ 75° with the opening facing the nest to allow the forager to climb out of the vegetation and back onto the foraging route and resume navigating. This procedure was repeated for training Trips 2–10.

After foragers completed their ten training trips, they began the testing phase and were no longer collected when passing through the site. As this testing was conducted to determine the persistence of the view memory’s current valance after the aversive outcome was removed and to characterize the number of experiences necessary to return back to pre-training baseline, we determined prior to the onset of the experiment that foragers would be tested for a total of five trips after training. On the forager’s eleventh trip to the feeder (Test 1), individuals were released from the feeder and allowed to travel the full inbound route to the nest without being collected and held (i.e. in the absence of the aversive outcome). As in training, foragers were recorded at Site 2 beginning just before they entered the grid. Given there was no collection during testing trips, recording ceased once foragers reached 25 cm past the grid at the end of the collection area. All of these foragers reached the nest and freely entered. On their next foraging trip, foragers were tested twice. These inbound foragers were recorded and allowed to pass through Site 2 (Test 2), identically to the previous test, until they reached the nest entrance. As foragers reached within 20 cm of the nest entrance with their path integrator now near zero and directionally uninformative (termed Zero Vector, ZV), each forager was picked up using an empty vial and returned to the foraging route 10 m from the nest (Fig. 1a). These foragers were allowed to resume their nest-ward journey and were recorded while passing through Site 2 identically to previous tests (Test 3 ZV) without the corresponding vector state present during training at these sites. Returning foragers collected at the nest are described as zero-vector as their path integrator (PI) no longer provides directional information to the nest, however note that the PI system is constantly in use and foragers in this test are still accumulating PI information. Importantly the foragers’ PI states during zero vector testing do not align with their PI states at the sites while training. During foragers’ next two foraging trips to the feeder their homeward journeys at Site 2 were recorded (Test 4 and Test 5) identically to Test 1. After Test 5 all testing on the individual ceased and foragers were collected at the nest, marked as tested and then released.

#### Hold duration tests

Next, we characterized whether foragers perceived differences in severity of aversive outcomes and responded differently to the associated views. Here, foragers (n = 14) were exposed to ten consecutive training trips where Site 1 was associated with a hold period of 300 s while Site 2 was associated with a hold period of 15 s (Fig. 2). A mirrored condition (Site 1–15 s, Site 2–300 s), was conducted on a second set of foragers (n = 14). Foragers were individually marked at the feeder and then released once they collected a food piece. During training (Trips 1–10), as foragers neared the grid at Site 1, they were recorded using the HD camera beginning just before the forager entered the grid area. As the forager exited the grid (8 m from the nest entrance), recording ceased and they were collected within a 25 cm area past the grid (grey area, Fig. 1a,b). Foragers were collected and held individually within the vegetation-filled vial for 300 s or 15 s (depending on condition) and then released back at the center of Site 1, using the procedure described in the previous experiment. After release, foragers were allowed to travel to Site 2 where they were again recorded within the grid at this site, then foragers were collected and held within the vegetation-filled vial upon exiting the grid (grey area, Fig. 1b). At Site 2, foragers were held for the other hold time before being released and then allowed to travel back to the nest with their food piece. On Trip 10, after release from Site 2, foragers were collected for testing as they reached the nest. These foragers were collected with no remaining vector (< 20 cm from the nest) using an empty vial and immediately released along the route 10 m from the nest (Fig. 2). Released foragers were allowed to return to the nest and were recorded while passing through the grid at both Site 1 and Site 2 without the corresponding vector state present during training at these sites. After testing, foragers were collected, marked as completed and released at the nest.

#### Risk sensitivity tests

In the final group of tests, we characterized foragers’ perceptions of fixed and risky aversive outcomes over 20 foraging trips. Here, for one set of foragers (n = 8), Site 1 was associated with a fixed aversive outcome, being held in vegetation for a period of 150 s on every training trip, while Site 2 was associated with a variable outcome, with a 50/50 chance of being held for a longer (300 s) or shorter (1 s) period. The arithmetic mean values of the Fixed and Risky site hold durations over training was chosen to be equal (~ 150 s), yet these training schedules had very different geometric means (Fixed Site, $\sqrt[1]{150}= 150$; Risky site, $\sqrt{1 \times 300}$ = 17.32). Given the short period within the vegetation during the 1 s hold time, special care was taken to confirm that this hold period did not start until foragers came in contact with the vegetation. A mirrored condition was conducted on a second group of foragers (n = 7) with these hold periods switched (Site 1–50/50 chance of a 300 s or 1 s hold period; Site 2–150 s hold period). Foragers were individually marked as they reached the feeder and then allowed to collect food and return towards the nest. At Site 1, foragers were recorded as they entered the grid then collected and held identically to previous conditions. After the designated holding period, foragers were released and allowed to travel to Site 2 where they were recorded as they entered the grid and then collected and held. This training occurred for 20 trips. After the Site 2 release on Trip 20, foragers were tested by being collected with a zero-vector state as they reached the nest, released at 10 m from the nest and their return trip through Site 1 and Site 2 was recorded without collection.

### Data digitization and analysis

Videos were digitized using GraphClick (Arizona Software). Each forager’s identity was coded prior to data collection so that the scoring was blind to the ant’s hold time condition. Paths were digitized by marking the ant’s mesosoma at 200 ms intervals beginning when the forager entered the grid and ceasing once foragers were collected during training or when they reached 25 cm past the grid edge during testing. Aversive view learning and memory were assessed by recording the number of hesitations exhibited by the forager leading up to collection. Two types of hesitation behavior were observed during testing, scans and stops and these were confirmed during video playback. We classified a ‘stop’ as a ceasing of forward movement with the ant remaining stationary until forward movement resumed. In contrast, ‘scans’ were classified as the ceasing of forward movement that is accompanied by the ant pirouetting, or rotating in place before resuming forward movement. Both of these behaviors were collected by the experimenter during the experiment and both positioning and behavior type was confirmed using video analysis. The quantity and position of both behaviors were recorded along the forager’s digitized paths. For statistical analysis, scan and stop behaviors were combined to create a total hesitation count for each training and test trip.

In the Aversive Learning tests, we compared hesitation numbers (Stops + Scans) across training/testing trips using a General Linear Model (GLM) for count data (Poisson loglinear) with individuals as a random effect. In the Hold Duration and Risk Perception tests, where foragers were collected for distinct hold periods at both Site 1 and Site 2, both *Site Number* and *Hold Condition* were analyzed as fixed effects. Conover’s post hoc pairwise comparisons of forager hesitations during the baseline during Trip 1 and after training/testing were conducted using p values corrected with the Bonferroni method for multiple comparisons (Aversive View Experiment, 14 comparisons; Hold Duration Experiment, 11 comparisons; Risk Sensitivity, 21 comparisons). All p values are presented post this correction (p × comparison #). Within individual comparisons between hold regimes in the *Hold Condition* (15 s vs. 300 s) and the *Risk Sensitivity* (Fixed vs. Risky) tests were compared using Wilcoxon Signed Rank Tests with p-values corrected with the Bonferroni method.

To further characterize the change in hesitation numbers at the Risky site, we calculated the change in hesitation (current hesitations minus hesitations on previous trip) number based on the outcome of the previous trip (held for 1 s or 300 s). For each individual forager, the mean hesitation change (excluding Trip 1) after a 300 s hold time was compared to the mean hesitation change after a 1 s hold time using a Wilcoxon Signed Rank Test.

#### Trip 10 comparisons

For between experiment comparisons, we chose to focus on forager hesitation numbers during training Trip 10 as, up to this point, the training schedule for each individual forager was consistent across all experiments. Across testing, we compared the Fixed and Risky site hesitation numbers to those of the 15 s site and 300 s hold conditions using Mann–Whitney *U* tests.

In a final analysis exploring why foragers in the Risky condition exhibited low hesitation numbers, we characterized the hesitation response by calculating a logarithmic curve of predicted hesitation numbers based on hesitations with captivity duration in abscise and hesitation number in ordinate. This curve of expected hesitations was calibrated using the hesitation numbers observed on Trip 10 in the 15 s hold condition in the Hold Duration experiment, the 150 s (Fixed) condition in the Risk Sensitivity experiment, and the 300 s conditions in the Aversive Views and Hold Duration experiments. We then compared the observed hesitations during Trip 10 of the Risky condition in the Risk Sensitivity experiment (μ = 3.33) to the predicted hesitations of the curve based on the Risky condition’s arithmetic mean hold time (150.5 s) and geometric mean hold time (17.32 s) using one sample T-tests to determine which hypothesis was favored.

## Results

### Aversive learning tests

**Figure 3:**
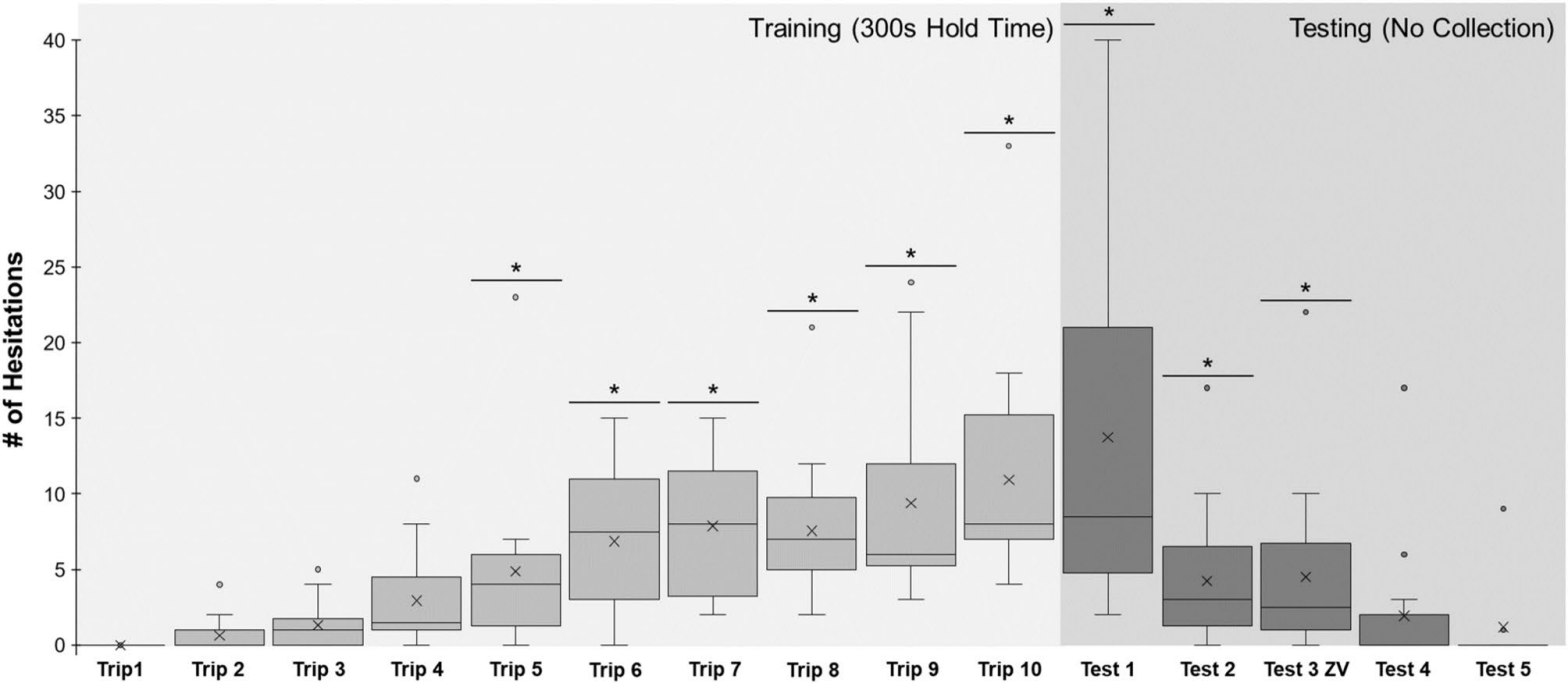
Forager hesitations during training and testing in the Aversive Learning tests. Foragers were collected at Site 2 during training (Trips 1–10, light grey), for 300 s and were then allowed to return to the foraging route. During testing (Test 1–5, dark grey), foragers were allowed to travel through Site 2 and return to the nest. Box plots show the median hesitations, consisting of scans and stops, across training and testing (middle line), mean hesitations (×) and 25th and 75th percentile (box) while the whiskers extend to min and max values (excluding outliers). Outliers were defined as values 150% of the IQR beyond 25th and 75th percentile and represented as individual points. *Denotes training or test trips where forager hesitations are significantly above the Trip 1 baseline (p < 0.05).

**Figure 4:**
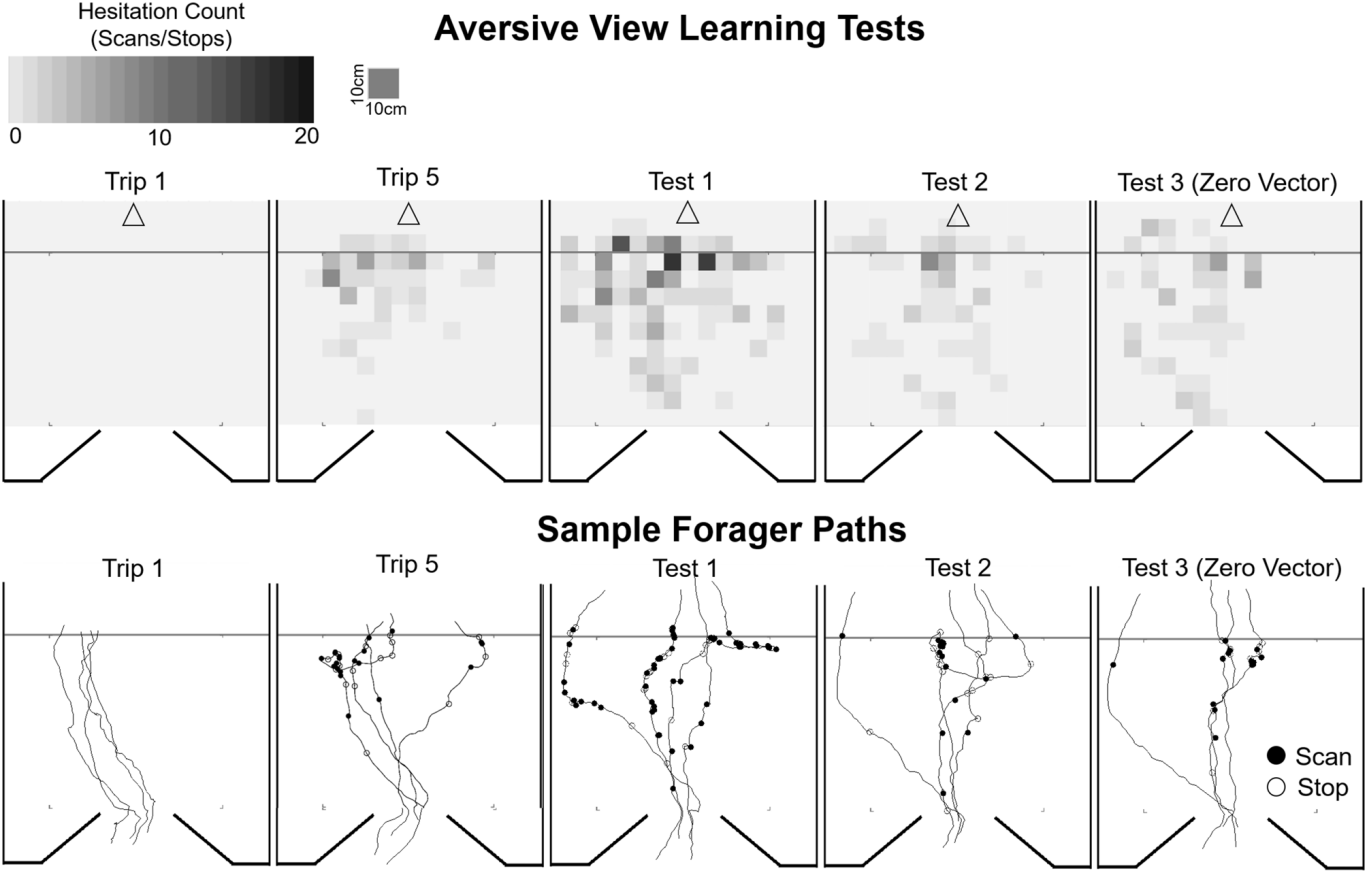
Heat maps of forager hesitation locations and sample paths in the Aversive Learning Tests. Heat maps of forager hesitations and five foragers’ sample paths during Trip 1, Trip 5, Test 1, Test 2 and Test 3. The nest direction is denoted by the arrow (top) for all panels and the black line crossing each heat map indicates the start of the collection area. Closed black circles denote locations of forager Scans while open circles denote Stop locations. For heat maps of hesitations for all training and test trips in the Aversive Learning tests, see SFig. 1.

Foragers traveling through Site 2 at the onset of training (Trip 1) showed no signs of hesitation leading up to the collection site (μ ± *S.E.* = 0.0 ± 0.0; Figs. 3, 4) and this was used as the baseline for hesitation comparisons during training and testing. *Trip Number* had a significant effect on foragers’ hesitation numbers (*Z* = 3.84; p < 0.001) with repeated exposures to 300 s captivity resulting in increased hesitations and this pattern increased as training progressed. Post hoc comparisons showed that hesitations did not significantly increase from the baseline on training trips 2–4 (p > 0.05; Figs. 3, 4). Beginning on Trip 5 (*T* = 3.52, p = 0.01) and continuing through the rest of training (Trip 6–10), hesitations were significantly higher than foragers’ baseline hesitation counts observed on Trip 1 (p < 0.001).

Hesitations remained significantly above baseline at the onset of testing when the aversive outcome was removed, however after multiple exposures to these views without collection, hesitations statistically returned to pre-training levels. During testing (Test 1–5), post hoc comparisons revealed that hesitation numbers remained significantly above baseline (Trip 1) during Test 1 (*T* = 6.39; p < 0.001), as well as two further trips after the aversive outcome was removed; Test 2 (*T* = 3.25*;* p = 0.021), and Test 3 ZV (*T* = 3.28; p = 0.019; Figs. 3, 4). Beginning on Test 4 (*T* = 1.46; p = 1.00) and continuing during Test 5 (*T* = 0.61; p = 1.00), hesitation counts returned to baseline and were not significantly different from Trip 1, suggesting that after three exposures to the site without the negative outcome, foragers’ level of aversion to the site had returned to pre-training levels. There was no significant difference between hesitation numbers during the last training trip, Trip 10 and Test 1 (*T* = 0.24*;* p = 1.00) as well as between Test 2 and Test 3 ZV (p = 0.87). Overall, these results suggest foragers exhibit evidence of aversive view learning after four prior experiences and these hesitations increased throughout training, peaking at Test 1 (μ ± *S.E.* = 13.8 ± 2.9). Evidence of aversive view memory retention persisted for two trips after the aversive outcome was removed (Test 2 and Test 3 ZV) before hesitations returned to baseline (Test 4 and Test 5; Fig. 3). Finally, the persistence of hesitations in zero vector ants confirm that the memories are associated, at least partially, with the views and not the forager’s vector state.

### Hold duration tests

**Figure 5:**
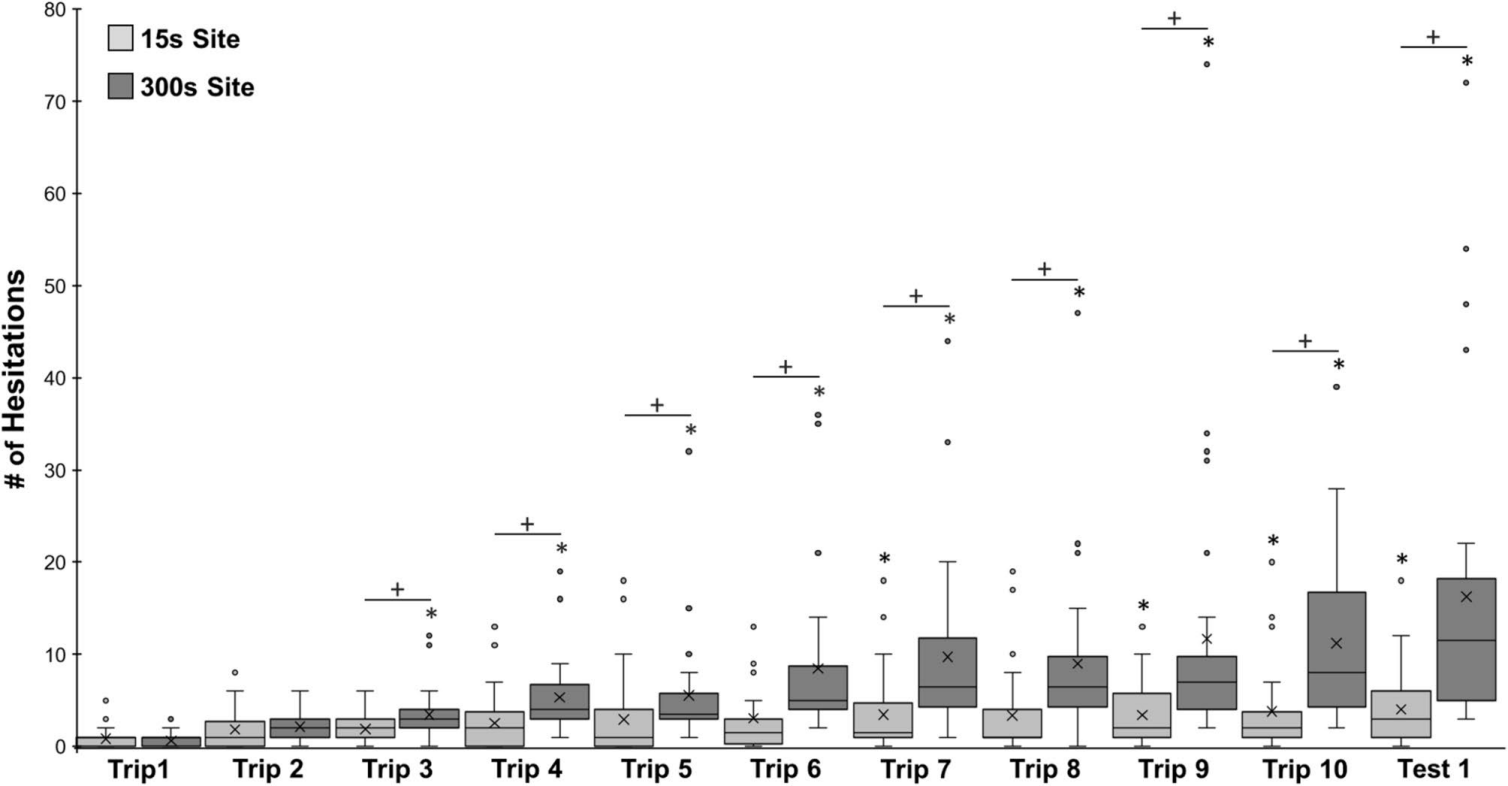
Forager hesitations during training Trips 1–10 and the zero vector test during Hold Duration tests. During training, foragers were collected at both Site 1 and Site 2 and held for either 15 s or 300 s before being released back at the site (hold periods for each site were randomly assigned at the onset of training for each individual). After training Trip 10, foragers were collected at the nest and tested as a ‘zero vector’ forager. During the test, foragers were allowed to travel through Site 1 and Site 2 without collection. Box plots show the median hesitations, consisting of scans and stops, across training and testing (middle line), mean hesitations (×) and 25th and 75th percentile (box) while the whiskers extend to min and max values (excluding outliers). Outliers were defined as values 150% of the IQR beyond 25th and 75th percentile and represented as individual points. Each *denotes training or test trips where foragers’ hesitation numbers were significantly above the Trip 1 baseline (p < 0.05). Each ‘+’ denotes trips in which the forager showed significantly higher hesitation numbers leading up to the 300 s collection site compared to the 15 s site (p < 0.05).

**Figure 6:**
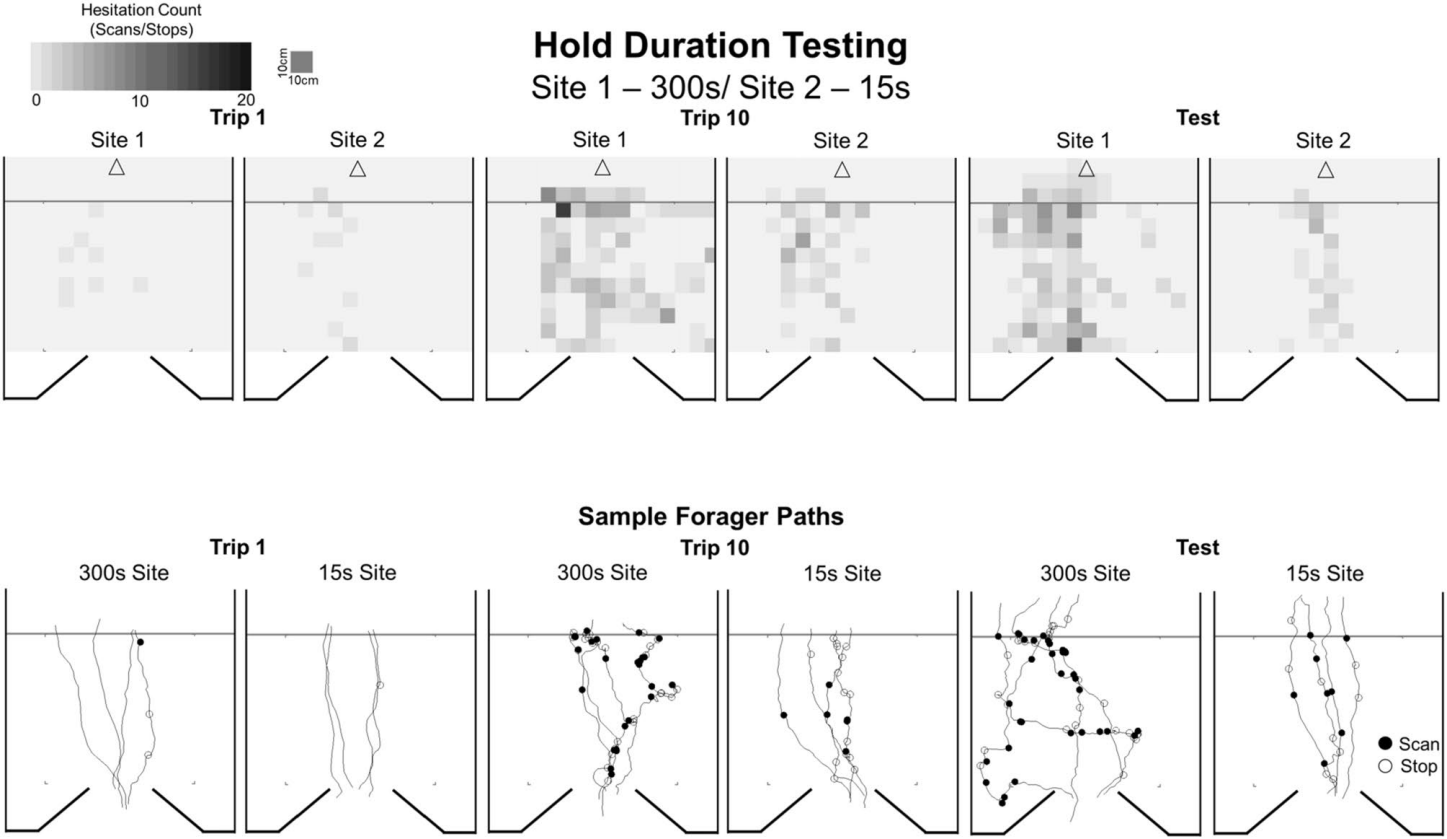
Heat maps of forager hesitation locations and sample paths in the Hold Duration testing. The nest direction is denoted by the arrow (top) for all panels and the black line crossing each heat map indicates the start of the collection area. Closed black circles denote locations of forager Scans while open circles denote Stop locations. For heat maps of hesitations for all training and test trips in the Hold Duration tests, see SFigs. 2 and 3.

Foragers at the onset of training (Trip 1) exhibited few pre-training hesitations leading up to both collection sites (15 s Trip 1, μ ± *S.E.* = 0.89 ± 0.21; 300 s Trip 1, μ ± *S.E.* = 0.64 ± 0.14; Figs. 5, 6) and these were used as the baselines for comparisons. Both *Trip Number* and *Hold Condition* had a significant effect on hesitations (*Trip Number*, *Z* = 6.21; p < 0.001; *Hold Condition*, *Z* =  − 3.47; p < 0.001) with repeated exposures to captivity resulting in increased hesitations and this pattern increased as training progressed with the two distinct hold times of 15 s and 300 s having a significant effect on these increases in hesitation numbers. Additionally, there was a significant interaction between *Trip Number* and *Hold Condition* (*Z* =  − 4.98; p < 0.001).

Finally, we found no difference in hesitation behaviour with regards to either the condition or training trip when hold times at Site 1 and Site 2 were mirrored. *Site Number* (condition mirroring) had no significant effect on forager hesitation numbers (*Z* = 0.34; p = 0.73) or significant interaction with either *Hold Condition* (*Z* = 1.05; p = 0.30) or *Trip Number* (*Z* = 0.85; p = 0.40), indicating that there was no difference between the sites or effect of the order foragers were exposed to the different hold times on aversive view learning.

At the 15 s hold associated site, foragers took six prior exposures to the outcome before a significant increase in hesitation behaviour was observed. Post hoc comparisons with the pre-training baseline showed that hesitations did not significantly increase on training Trips 2–6 (p > 0.05). Increased hesitation behaviour was first observed on training Trip 7 (*T* = 3.11; p = 0.022). However, during Trip 8 the Bonferroni adjusted p-value of the baseline hesitation comparison (*T* = 2.84, p = 0.055), fell just above (p = 0.05) the significance threshold. Hesitation counts were again significantly above baseline for the final two training trips (Trip 9, *T* = 3.02; p = 0.043; Trip 10, *T* = 3.88; p < 0.001) as well as the zero vector Test (*T* = 3.69; p < 0.001). In contrast at the 300 s hold site, post hoc comparisons showed that foragers learned the association after only two exposures, with hesitations significantly above Trip 1’s baseline beginning on Trip 3 (*T* = 3.41; p = 0.01). This significant increase in hesitations persisted through the rest of training on Trips 4–10 as well as the zero vector Test (p < 0.001). Comparisons between the final training trip, Trip 10, and the zero vector Test showed no significant difference in hesitations at either the 15 s site (*T* = 0.19; p = 1.00) or 300 s site (*T* = 1.14; p = 1.00).

Comparisons of forager hesitation numbers between the 15 s and 300 s sites showed that before training (Trip 1) foragers showed no significant differences between Site 1 and Site 2 (Wilcoxon signed-rank, Z = 43.5, p = 1.00). Foragers also showed no significant increase in their hesitations at the 300 s site during Trip 2 (p = 0.23). Beginning on Trip 3 (p = 0.002), and continuing for the rest of training (Trips 4–10), foragers exhibited significantly higher hesitations at the 300 s hold site compared to the 15 s hold site (p < 0.005) and this difference persisted during the zero vector Test (p < 0.001). Foragers were able to perceive differences in outcome severity between two hold times, as foragers learned the association at the 300 s hold site faster than the 15 s site (Trip 3 vs. Trip 7) and showed higher hesitation counts associated with the more severe outcome associated site.

### Risk sensitivity tests

**Figure 7:**
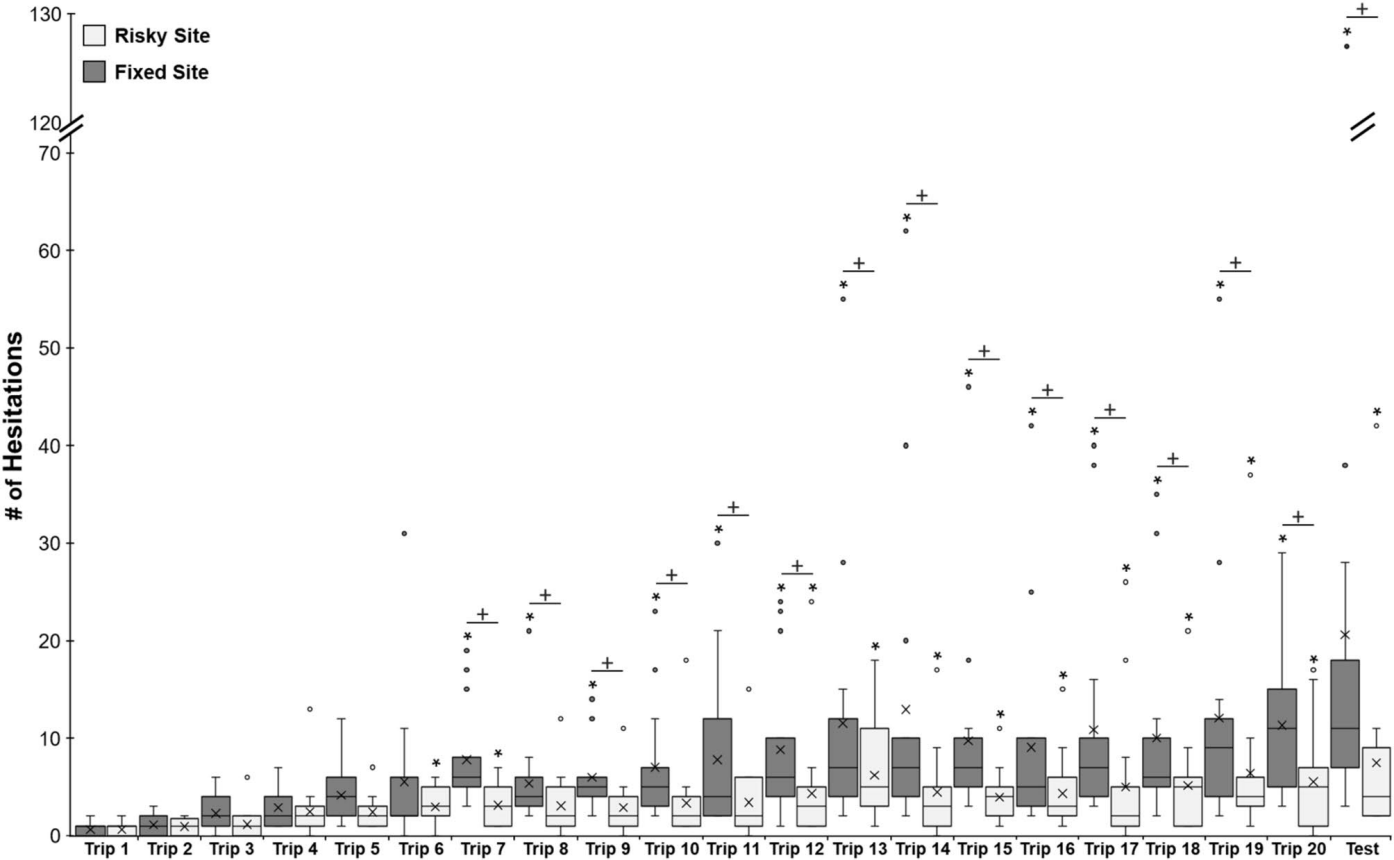
Forager hesitations during training Trips 1–20 and one zero vector test in the Risk sensitivity tests. During training, foragers were collected at both Site 1 and Site 2 and held within a vegetation-filled container for either a fixed or risky period. At the fixed site, foragers were held for 150 s, while at the risky site foragers had a 50/50 chance of being held for 1 s or 300 s. After the hold period, foragers were allowed to climb out of the container and return with their food to the nest. After training Trip 20, foragers were collected at the nest and tested as ‘zero vector’ foragers, by placing them back onto the route at 10 m from the nest. During the test, released foragers were allowed to travel through Site 1 and Site 2 without collection. Box plots show the median hesitations, consisting of scans and stops, across training and testing (middle line), mean hesitations (×) and 25th and 75th percentile (box) while the whiskers extend to min and max values (excluding outliers). Outliers were defined as values 150% of the IQR beyond 25th and 75th percentile and represented as individual points. Each *Denotes training or test trips where forager hesitation numbers were significantly above the Trip 1 baseline (p < 0.05). Each ‘+’ denotes trips in which the forager showed significantly higher hesitation numbers leading up to the fixed (150 s) collection site compared to the risky (1 s/300 s) site (p < 0.05). For heat maps of hesitations for all training and test trips in the Risky Perception tests, see SFigs. 4 and 5.

As in previous conditions, foragers at the onset of training (Trip 1) in both the Fixed and Risky conditions exhibited few hesitations leading up to the collection sites (Fixed Trip 1, μ ± *S.E.* = 0.60 ± 0.16; Risky Trip 1, μ ± *S.E.* = 0.69 ± 0.18; Fig. 7) and this was used as the baseline for future comparisons. Both *Trip Number* and *Hold Condition* (classified as Fixed or Risky) had a significant effect on hesitations (*Trip Number*, *Z* = 6.41; p < 0.001; *Hold Condition*, *Z* = 3.54; p < 0.001; Fig. 7) with repeated exposures to captivity resulting in increased hesitations and this pattern increased as training progressed with the two distinct hold schedules (Fixed and Risky) having a significant effect on these increases in hesitation numbers. There was no significant interaction between *Trip Number* and *Hold Condition* (*Z* = 1.1; p = 0.24).

Lastly, we found no difference in hesitation behaviour with regards to either the condition or training trip when hold times at Site 1 and Site 2 were mirrored. *Site Number* (condition mirroring) had no significant effect on forager hesitation numbers (*Z* = 0.47; p = 0.64) or significant interaction with *Hold Condition* (*Z* = 0.34; p = 0.73) or *Trip Number* (*Z* = 0.91; p = 0.36). This indicates that there was no difference between the sites or effect of the order foragers were exposed to the different hold times on aversive view learning.

At the Fixed site, post hoc comparisons showed that hesitations did not significantly increase from the baseline on training Trips 2–6 (p > 0.05; Fig. 7). On Trip 7, hesitations were significantly higher than foragers’ baseline hesitation counts (p = 0.002) and this significant hesitation increase persisted throughout the rest of training on Trips 8–20 (p < 0.05; Fig. 7) During the zero vector Test, forager hesitations were also significantly above baseline (p < 0.001).

At the Risky site, post hoc comparisons showed that hesitations did not significantly increase from the baseline on training Trips 2–5 (p > 0.05; Fig. 7). Hesitations were significantly above baseline during training Trip 6 (p = 0.02) and Trip 7 (p = 0.02), but not during Trip 8–11 (p > 0.05). Beginning on Trip 12, hesitations were again significantly above baseline (p = 0.02) and this difference persisted through the rest of training (p < 0.05). During the Test, ZV forager hesitations were also significantly above baseline (p = 0.01; Fig. 7).

Comparisons of forager hesitations between fixed and risky hold schedules showed that, before training (Trip 1), foragers showed no significant differences between Site 1 and Site 2 (Wilcoxon signed-rank; Z = 10.50; p = 1.00). During training, foragers also showed no significant difference in their hesitations between sites during Trip 2 (Wilcoxon signed-rank; Z = 32.5; p = 1.00) and this persisted through Trip 6 (Wilcoxon signed-rank; Z = 11.02; p = 0.20). Beginning on Trip 7 (Wilcoxon signed-rank; Z = 5.01; p = 0.004) and continuing through the rest of training (Trips 8–20), foragers exhibited significantly higher hesitations at the Fixed hold site compared to the Risky hold site (p < 0.05) and this difference was also present during the zero vector Test (p < 0.001).

**Figure 8:**
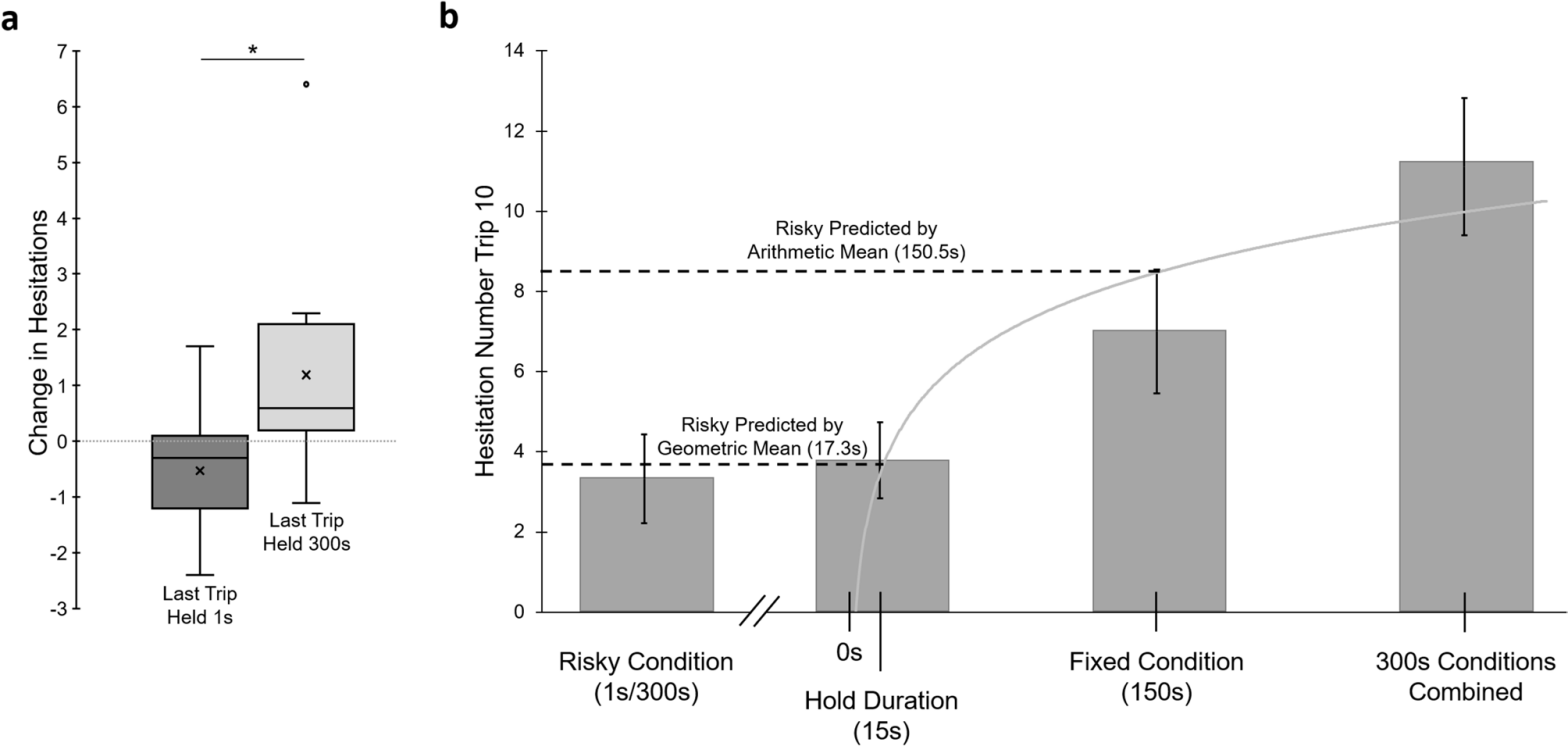
Hesitation changes by last experienced outcome in the Risky condition and between condition comparisons on training Trip 10. (**a**) Mean Change in hesitation numbers in the Risky condition across training based on the outcome of the previous trip (Held 300 s or 1 s). Mean change across training was calculated per individual. Box plots shows the median hesitation change (middle line), mean hesitation change (×) and 25th and 75th percentile (box) while the whiskers extend to min and max values (excluding outliers). Outliers were defined as values 150% of the IQR beyond 25th and 75th percentile and represented as individual points. *Denotes a significant difference between conditions (p < 0.05). (**b**) Predicted hesitations at training Trip 10 along a logarithmic curve (grey line) calibrated by hesitation numbers in the Hold Duration (15 s), Fixed condition (150 s), and the combined 300 s conditions (mean hesitations ± *S.E.*). The Risky condition’s mean number of hesitations on Trip 10 is plotted on the left, with the predicted value of either the condition’s geometric mean hold time of 17.3 s or its arithmetic mean hold time of 150.5 s. The observed hesitations during Trip 10 of the Risky condition (μ ± *S.E* = 3.3 ± 1.1) fell well below the predicted hesitation number given the arithmetic mean hold time (3.33 observed vs. 8.5 predicted) yet the logarithmic curve of predicted hesitations (3.8) falls within the standard error of the observed hesitations at the condition’s geometric mean. The observed lower hesitations during risky training aligns with the principle that the forager’s perception, and resulting hesitation behavior, of the aversive outcome has a logarithmic relationship with stimulus strength (y = 2.182 × ln(x) − 2.465).

During training at the Risky site, changes in hesitation numbers suggest that the effect of training was continuously regulating hesitation behavior up and down based upon differences in the expected outcome and forager’s experience on each trip (Fig. 8a). After experiencing the highly aversive 300 s outcome, hesitations are regulated upward (mean hesitation change ± *S.E.* =  + 1.19 ± 0.44) while experiencing the less aversive 1 s outcome resulted in hesitations being regulated downward (mean hesitation change ± *S.E.* =  − 0.53 ± 0.29) and these changes based on the last experience were significant (Wilcoxon signed-rank; Z =  − 2.50; p = 0.012).

### Between condition comparisons

During Trip 10, forager hesitations in the two 300 s conditions (the Aversive Learning tests and Hold Duration tests) showed no significant differences (Mann–Whitney U; U = 210.5; p = 0.37) and these data were combined for future comparisons. Hesitation numbers during the Fixed 150 s condition were significantly lower than the 300 s conditions (Mann–Whitney U; U = 196.5; p = 0.048) and significantly higher than the 15 s condition (Mann–Whitney U; U = 196.5; p = 0.002)*.* In contrast, forager hesitations in the Risky condition, (mean hold time = 150.5 s) were not significantly higher from the hesitation numbers of foragers in the 15 s condition of the Hold Duration tests (Mann–Whitney U; U = 196.5; p = 0.741) despite the order of magnitude difference in mean hold time (150.5 s vs. 15 s; Fig. 8b).

Finally, observed hesitations during Trip 10 of the Risky condition (μ = 3.33) were compared along the logarithmic curve of predicted hesitation numbers, calibrated by the other conditions (y = 2.182 × ln(x) − 2.465; R = 0.9293) at both its arithmetic (150.5 s) and geometric (17.32 s) mean hold times. At Trip 10, observed hesitations in the Risky condition were not significantly different from the predicted hesitation number (3.75) based upon the geometric mean hold time of 17.32 s (One sample T-test; *T* = 0.377; p = 0.712; Fig. 8b). In contrast, these observed hesitations significantly differed from the predicted hesitation number (8.53) based upon the arithmetic mean hold time of 150.5 s (One sample T-test; *T* = 4.67; p < 0.001).

## Discussion

In all tests, foragers traveling through the grid leading to their first collection exhibited either no or minimal hesitation behaviours. Hesitations, including scanning behaviours, typically occur when there are increases in navigational uncertainty due to inexperience, cue conflicts or decreases in view familiarity^32^. Given the high likelihood that foragers had multiple experiences of the route during previous successful foraging trips to the feeder, it was expected that hesitation numbers during Trip 1 would be low. When foragers were trained at either site along the homeward route with a captivity period in the vegetation-filled container, these individuals learned an association between the views preceding the collection site and the outcome, showing a significant increase in hesitation numbers when encountering these views on the following trips. Foragers retained these view-based associations even after the outcome was removed for multiple trips. Trained foragers continued to hesitate above their baseline on the next three trips where they were allowed to pass through without collection, suggesting the behavioral response was not based solely on the previous foraging trip. Foragers tested without a corresponding vector state showed no change in hesitation numbers, meaning the association was tied primarily to the view memory and not an association between the vector state and outcome.

### Aversive view learning

Models of visual navigation currently rely solely on the positive valence, or attractiveness, of familiar views which inhibit search behaviour (turning) and induce forward movement^13,24,25,53^. These positive valance memories involve reinforcement learning of the associated inbound views that lead to the nest, likely reinforced upon the forager’s arrival, though the exact reinforcer remains unknown. Yet, recent work has demonstrated that views can also be associated with negative outcomes leading these views to develop a negative valence, or aversiveness, which inhibits forward movement and induces hesitations, turns and scanning behaviour^31^. Such behaviours increase the likelihood that foragers may avoid the negative outcome experienced on the old route and return to the nest quickly along a new route, leading to these new views developing a positive valance and the formation of detours^27,28,31^.

In the current study, just as in Wystrach et al.^31^, our results show rapid acquisition of aversive view memories at specific spatial locations associated with negative outcomes, resulting in increased hesitations leading up to these sites. Views that previously had a positive association, formed during the initial formation of the homeward route, subsequently become negative when associated with the experience of struggling within the vegetation. Unlike previous work, here foragers were unable to form new positively reinforced routes detouring around these negative outcomes as all available homeward routes resulted in collection. Once the negative outcome was removed, foragers were shown to take two exposures to the route to re-learn its positive association, reinforced through re-entering the nest (during Test 1 and Test 3 ZV), and reduce hesitations to baseline. As Test 2 did not result in the forager successfully entering the nest (due to collection for Test 3 ZV testing), it is likely the observed lack of a decrease in hesitations between Test 2 and Test 3 (Fig. 3) was influenced by the missing reinforcement of re-entering to the nest, rather than reaching a zero-vector state or experiencing the nest panorama. This result hints that positive reinforcement of the route views upon a successful foraging trip may trigger only once the forager enters the nest. Clearly further characterization of the reinforcement learning that occurs during route formation is warranted.

### Outcome severity and risk perception

In associating views with negative outcomes, foragers are able to distinguish between levels of severity of outcome, which was evident both in the acquisition rates and overall hesitation responses. Foragers in the Hold Duration tests rapidly learned the association at the 300 s site, showing increased hesitation behaviour after only two previous exposures. In contrast, these same foragers required six exposures to show increased hesitation behavior at the 15 s site. Overall hesitations also differed by outcome severity, with foragers exhibiting significantly more hesitations associated with the 300 s site (μ = 11.7) than the 15 s site (μ = 3.8) during the final training trip (Trip 10). In contrast, foragers trained in the Risk Perception tests at the fixed 150 s site, exhibited a hesitation response mid-point between these two extremes (μ = 7.3). These differences suggest foragers were able to perceive time disparities spent struggling within the vegetation and recalled this outcome severity on subsequent foraging trips, leading to distinct levels of aversion behavior expressed at each site.

Risk variance did not affect how quickly foragers learned the negative association, but had a significant effect on the degree of hesitation that developed. Over the final five training trips, foragers exhibited twice the number of hesitations at sites that resulted in a fixed negative outcome (150 s; μ = 10.7) compared to sites leading to risky outcomes (1 s or 300 s; μ = 5.3) suggesting foragers perceived the risky outcome as less severe than the fixed outcome despite these schedules having the same mean hold time over the course of training (~ 150 s). This increased hesitation number at the fixed site versus the risky site persisted during the test (μ = 20.6 and 7.9 respectively). Furthermore, hesitation number at the risky site was shown to increase or decrease based upon the outcome experienced on the forager’s previous trip. When foragers experienced a severe 300 s hold time on the preceding trip, hesitations increased (μ =  + 1.16) while when the less aversive 1 s hold time was experienced on the preceding trip, hesitations decreased (μ =  − 0.51). These changes indicate that forager’s hesitancy to pass through the site is being continuously regulated up or down with each new experience of the site. During each training trip, the hesitation behavior expressed represents the forager’s level of aversion to the expected outcome while each new experience regulates this expectation.

Additionally, the hesitation data presented here shows that foragers’ perception of risky aversive outcomes was not optimal in terms of the true value of the outcome. Many studies of preference variance in reward quality/quantity show a general risk aversion tied to the animal’s perception of rewards balanced only by mean^36^. This has been demonstrated in ants, as De Agrò et al.^43^ showed that ant foragers were risk averse when reward options were balance by mean value. Yet, fixed option preference disappeared when the two reward options were altered to be geometrically balanced. This finding makes sense if animals perceive reward value on a logarithmic scale. For positive outcomes, when the geometric average of a risky reward option falls below the fixed reward option, animals should perceive the fixed option as preferable. Our research suggests that this relationship also fits with the study of variability of negative outcomes. When the geometric average of a negative outcome falls below the fixed outcome, animals should perceive this risky outcome as less severe and become risk seeking, which aligns with our results. In the Risk Sensitivity tests, the two outcomes were only balanced by true value (150 s vs. 150.5 s), while the geometrical average of the hold duration of the Risky site (17.32) was lower than the Fixed site (150). This corresponded with an overall lower level of hesitations at the Risky site compared to the Fixed site. While we did not test purely geometrically balanced Risky vs. Fixed outcomes, the perceived stimulus strength of the Risky site based on the geometrical average would make it very similar to that of the 15 s outcome in the Hold Duration tests (17.32 s vs. 15 s). Interestingly, the hesitation levels during training (Trip 10) between the 15 s outcome (μ = 3.8) and the Risky outcome (μ = 3.3) were not significantly different from one another despite the order of magnitude difference in actual mean hold duration (150.5 s vs. 15 s; Fig. 8b). Additionally, we calculated a logarithmic curve of expected hesitations based on those observed during the 15 s, 150 s and 300 s conditions on Trip 10 (Fig. 8b). When hesitations observed during Trip 10 at the Risky site were compared with this curve, observed and predicted hesitations did not significantly differ at the geometric mean (17.32 s) but did significantly differ from the predicted hesitations at the Risky condition’s arithmetical mean (150.5 s), demonstrating that the geometric mean hypothesis should be favored over the arithmetic mean. This provides further evidence that there is a logarithmic relationship between captivity duration and forager’s response, suggesting foragers are perceiving the outcome severity logarithmically rather than its true value.

Stimulus strength perception is typically confirmed by animal’s choices of varying rewards and is used to explain why animals are typically risk averse to variable rewards^41^. The current results indicate that such factors also predict risk seeking behavior to negative outcomes. Here, the foragers faced with two identical mean hold times perceived the variable outcome as less severe, and equal to a hold time almost an order of magnitude lower than its true value (17.3 s vs. 150.5 s) and thus respond less negatively to the associated views, on par with hesitations to a fixed 15 s hold time (Fig. 8b). In our Risk Perception testing, the constant site always results in a 150 s hold while the risky site may result in a 1 s or 300 s hold time. Along a logarithmic curve, this 150 s hold time would be perceived by the forager as 150 times worse than the 1 s hold time while 300 s is only two times worse than 150 s. If these outcomes were balanced by geometric mean rather than true mean, for example altering the hold times to a constant 15 s hold time and a risky schedule of a 50% chance of either 1 s or 225 s ($\sqrt{1 \times 225}$ = 15), we would expect the forager’s perception of the aversive outcomes to be equal and result in identical hesitation numbers. While the current findings clearly point to the logarithmic relationship between the outcome’s severity and the forager’s aversive response, future work could help further untangle the ant’s perception of aversive outcomes, effort and risk by testing truly geometrically balanced fixed and variable outcomes.

### Underlying neural mechanisms

Our understanding of the neural underpinnings of insect navigation has tremendously increased in the last decade^52^, and notably, regarding how views are memorised and associated to specific valence in the insects' mushroom body^53–58^. It is therefore possible and useful to see whether and how our current behavioural results can be interpreted in the light of this neural framework. While some of our results can be readily explained by our current neural understanding, others bear new implications, and can help us derive novel predictions at the neural level.

The use of learned route memory in ants involves the Mushroom bodies, MBs^55,56^, and its known neural circuitry can explain the storage and recall of visual as well as olfactory memories^31,54,57,58^. Visual information enters the MBs via projection neurons from the optic lobes^59^. An individual view can be represented neurally within the MBs through activation patterns of Kenyon Cells (KC), which project onto a number of motor output neurons (MBON). Each MBON conveys an attractive or aversive valence, and changes in synaptic strength between KCs and MBONs by activation of dopaminergic neurons mediating negative or positive experiences, modulates the association between a stimulus and its outputted valence^60,61^. Changes within these synaptic compartments mediate the view’s current overall valence by weighting the attractive and aversive valences of the forager’s accumulated experiences. More specifically, the aversive outcome of being captured, as in our experiment, must result in dopaminergic neurons decreasing the connection strength between the recently encountered view specific pattern of Kenyon cells and attractive valence MBONs^31^. Such a neural architecture can explain the hesitation behaviours observed after training foragers with the aversive outcome.

The current findings support our understanding of the memory dynamics within the circuity of the MBs, First, the aversive response increased across repeated trial until reaching a plateau (Fig. 7). This echoes what is observed in olfactory conditioning in other insects^62^. Second, while aversively trained foragers were highly hesitant to travel through these sites, no forager refused to cross the line, even when they lacked information from a path integration based vector (Figs. 4, 5). This suggests that both attractive and aversive valence memory traces are simultaneously at play. Neurally, the co-existence of attractive and aversive valences suggests that their memory traces persists in different MBONs, as demonstrated in fly’s MBs for olfaction^63^. Third, the sequential changes in hesitation number in the risky condition indicate that forager behaviour is being continuously regulated based by each new trip’s experienced outcome (Fig. 8a). Thus, current experience continuously regulates the forager’s expected outcome at the site, as observed in the fly’s MBs^60^. However, the persistence of hesitations after the aversive outcome was removed show that valence likely persists, and is thus the result from an accumulation of experiences over multiple previous trips, not the last experience alone. Fourth, the reduction in hesitation following a 1 s hold time in the risky condition (Fig. 7) shows that this experience led to a net gain in positive valence, even though the ant has been captured. This supports the idea that learning in the MB follows a prediction-error rule which has also been demonstrated in flies^64^. Learning is dependent on the discrepancy between the current experience and the expected one^65^. In other words, being captured for 1 s at a site where one has been previously captured for 300 s mediates a positive reinforcement, leading to a decrease in aversion. Overall, these behavioral results suggest that ants’ MBs in the context of visual learning may work very similarly to those of *Drosophila* for olfactory learning^64^ A similar memory dynamic framework would mean that within the ant’s MBs various dopaminergic neurons continuously modulates connection strength of various aversive and attractive valence MBONs based on the difference between the expected outcome and the experienced outcome on that trip.

## Conclusions

We found that *C. velox* foragers rapidly learn to associate views with aversive outcomes, showing increased hesitations at these sites, in some cases after only two previous experiences. Such memories are not solely based on the forager’s most recent trip, as individuals continued to showed increased hesitation at these sites after the aversive outcome was removed, suggesting these aversive memories persist over multiple trips. Foragers were also able to perceive differences in outcome severity, learning more rapidly and exhibiting more hesitations at a site associated with a severe outcome (300 s) when compared to a less severe outcome (15 s). Additionally, we show that the foragers show significantly less apprehension to travel through a site associated with a risky aversive outcome compared to a fixed outcome with the same mean and that forager hesitation responses at these sites across experiments was in line with the logarithmic relationship between stimulus strength and perception. Finally, our findings fit within the current modeling of view-based route learning and memory in the mushroom bodies of the insect brain. The behavioral dynamics observed here align well with the complex and parallel memory dynamics of the MB as studied in the context of olfaction in flies. In closing, a final intriguing question remains. Namely, the foragers’ response to different hold durations suggests that they can somehow quantify or estimate their duration of capture, yet the mechanism by which this estimate over such short time scales (~ 135 s) is accomplished currently remains unknown.

## Supplementary Information

Supplementary Information.

## Data Availability

The datasets analysed during the current study are available online from: 10.13140/RG.2.2.29988.40321.
