# Supplementary material for: Aversive view memories and risk perception in navigating ants
Source: Sci Rep. 2022 Feb 21;12:2899. doi: 10.1038/s41598-022-06859-4 (PMC8861035; doi:10.1038/s41598-022-06859-4)
Supplement: Supplementary file 1 — Supplementary Information. [file 41598_2022_6859_MOESM1_ESM.pdf]

Aversive View Memories and Risk Perception in Navigating Ants

Cody A Freas*[1], Antoine Wystrach[2], Sebastian Schwarz[2], Marcia L Spetch[1]

[1]Department of Psychology, University of Alberta, Edmonton, Alberta, Canada

[2] Research Center on Animal Cognition (CRCA), Center for Integrative Biology (CBI), CNRS, University Toulouse III-Paul Sabatier, Toulouse, France

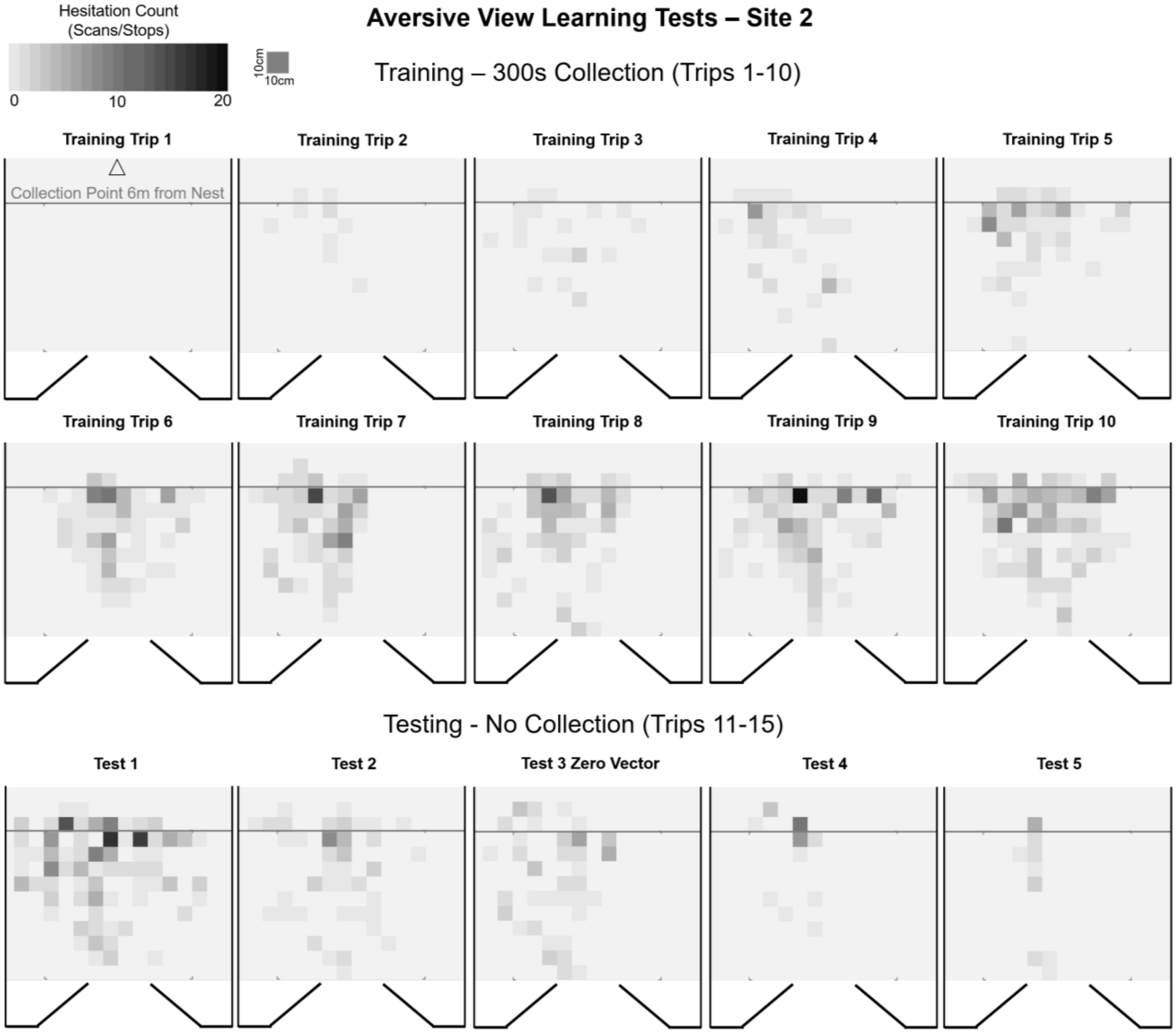

Supplemental Fig. 1. Heat maps of forager hesitation locations at Site 2 in the Aversive Learning Tests. During training Trips 1–10, foragers (n = 16) were collected and held for 300sec after they exited the testing grid (grey line, Collection Point) 6m from the nest entrance. The nest direction is denoted by the arrow (top). During Test 1–5, foragers were not collected and instead allowed to travel through Site 2 and to the nest entrance. Foragers were tested with no corresponding homeward vector during Test 3 (Zero Vector).

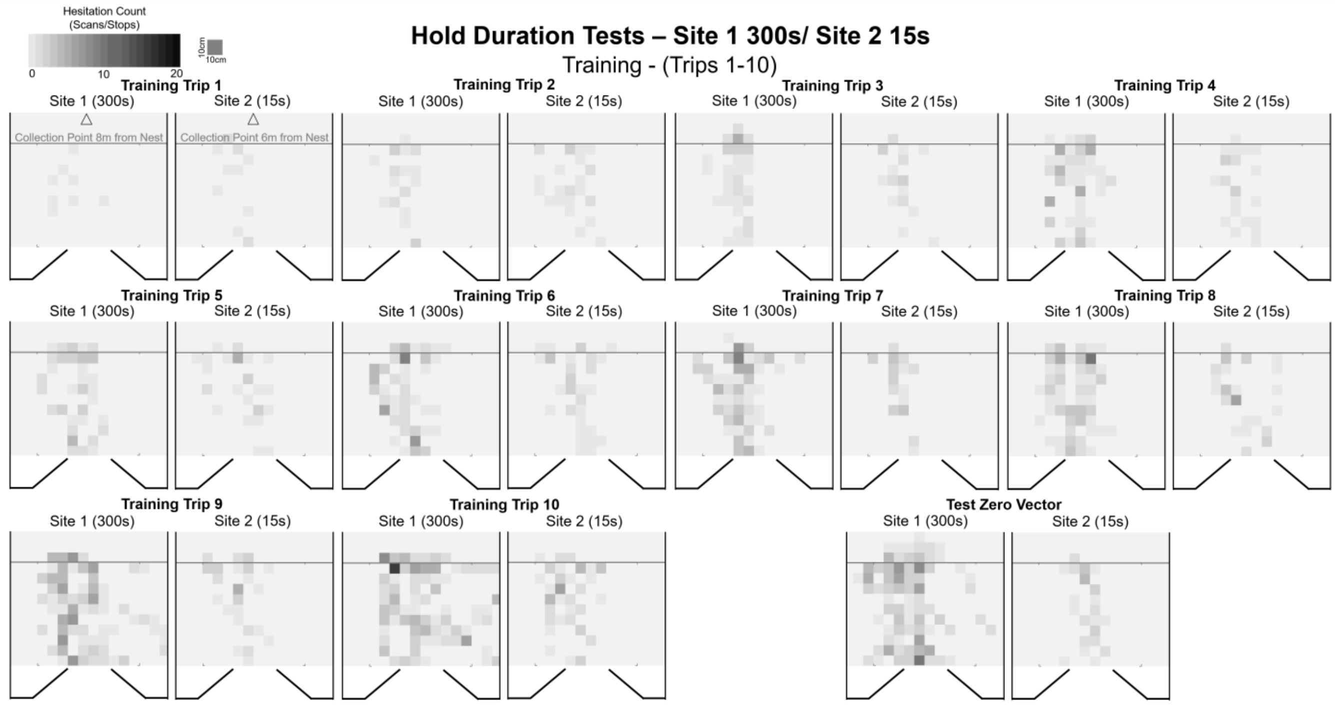

Supplemental Fig. 2. Heat maps of forager hesitation locations in the Hold Duration Tests (Site 1 – 300sec / Site 2 – 15sec). Here, Site 1 was associated with a 300sec hold time and Site 2 with a 15sec hold time. During training Trips 1–10, foragers (n = 14) were collected after they exited the testing grid (grey line, Collection Point) at both Site 1 and 2 (8m and 6m from the nest entrance respectively). The nest direction is denoted by the arrow (top). During the Test Trip, foragers were tested with no corresponding homeward vector (Zero Vector) and allowed to pass through both sites and travel to the nest entrance.

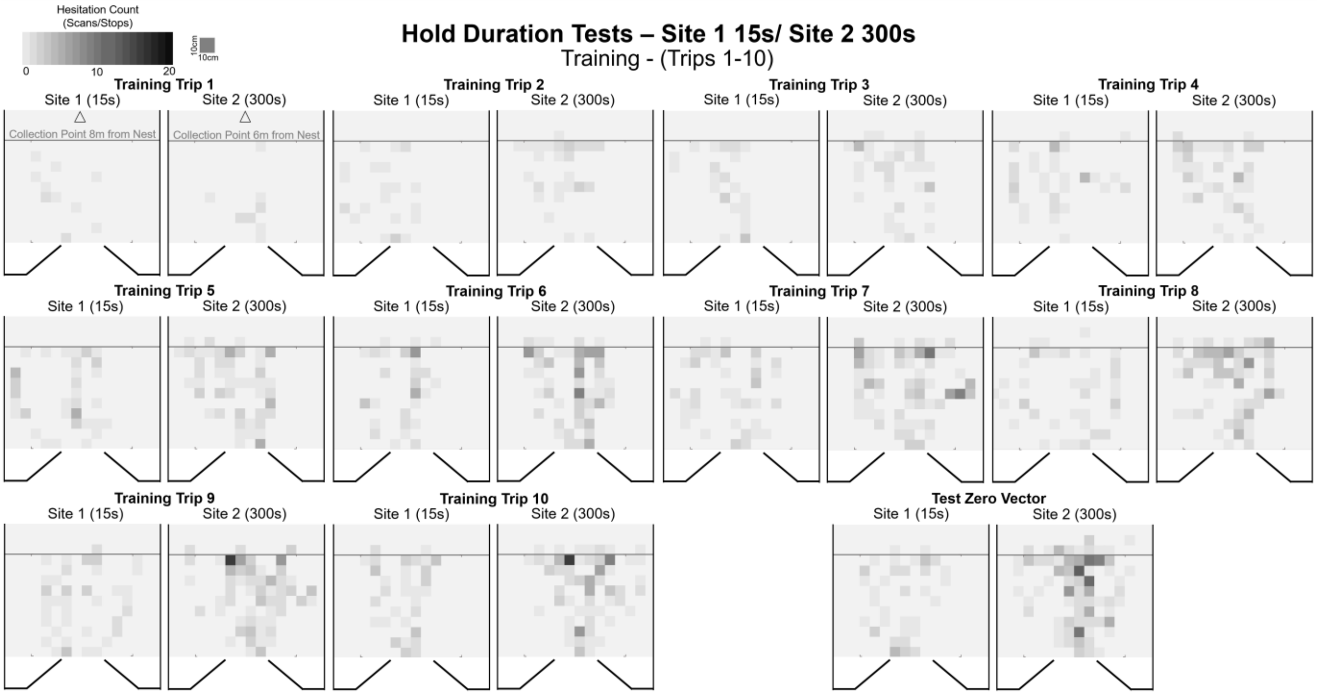

Supplemental Fig. 3. Heat maps of forager hesitation locations in the Hold Duration Tests (Site 1 – 15sec / Site 2 – 300sec). Here, Site 1 was associated with a 15sec hold time and Site 2 with a 300sec hold time. During training Trips 1–10, foragers (n = 14) were collected after they exited the testing grid (grey line, Collection Point) at both Site 1 and 2 (8m and 6m from the nest entrance respectively). The nest direction is denoted by the arrow (top). During the Test Trip, foragers were tested with no corresponding homeward vector (Zero Vector) and allowed to pass through both sites and travel to the nest entrance.

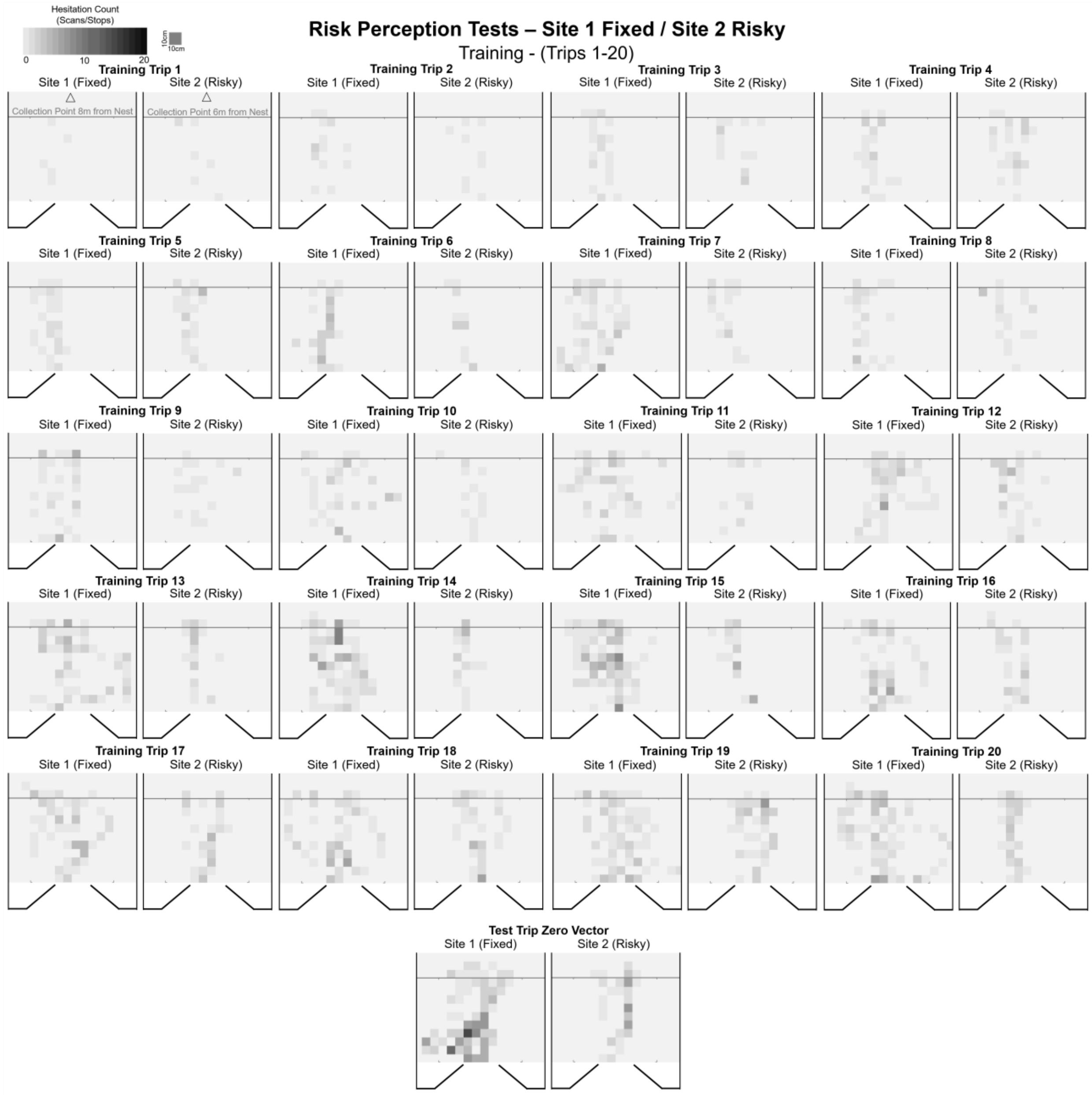

Supplemental Fig. 4. Heat maps of forager hesitation locations in the Risk Perception Tests (Site 1 – Fixed / Site 2 – Risky). Here, Site 1 was associated with a fixed 150sec hold time and Site 2 with a 50% chance of either a 1sec or 300sec hold time. During training Trips 1–20, foragers (n = 8) were collected after they exited the testing grid (grey line, Collection Point) at both Site 1 and 2 (8m and 6m from the nest entrance respectively). The nest direction is denoted by the arrow (top). During the Test Trip, foragers were tested with no corresponding homeward vector (Zero Vector) and allowed to pass through both sites and travel to the nest entrance.

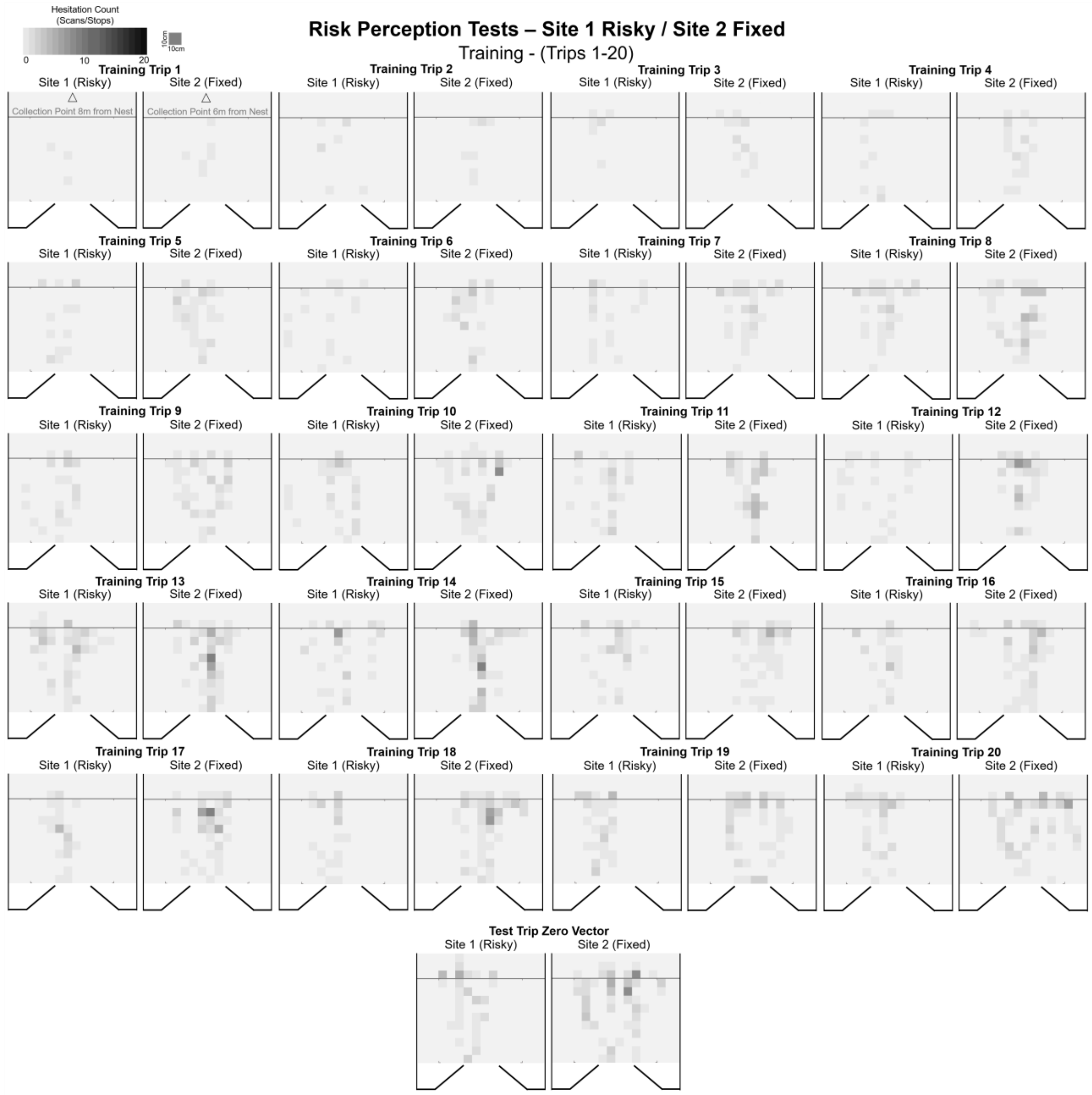

Supplemental Fig. 5. Heat maps of forager hesitation locations in the Risk Perception Tests (Site 1 – Risky/ Site 2 – Fixed). Here, Site 1 was associated with a 50% chance of either a 1sec or 300sec hold time and Site 2 was associated with a fixed 150sec hold time. During training Trips 1–20, foragers (n = 7) were collected after they exited the testing grid (grey line, Collection Point) at both Site 1 and 2 (8m and 6m from the nest entrance respectively). The nest direction is denoted by the arrow (top). During the Test Trip, foragers were tested with no corresponding homeward vector (Zero Vector) and allowed to pass through both sites and travel to the nest entrance.
